# Childhood trauma and childhood urbanicity in relation to psychotic disorder

**DOI:** 10.1007/s00127-015-1049-7

**Published:** 2015-04-21

**Authors:** Aleida Frissen, Ritsaert Lieverse, Marjan Drukker, Ruud van Winkel, Philippe Delespaul, GROUP Investigators

**Affiliations:** Department of Psychiatry and Psychology, Maastricht University, PO Box 616 (DRT10), 6200 MD Maastricht, The Netherlands

**Keywords:** Psychosis, Childhood, Trauma, Urbanicity, Environment

## Abstract

**Background:**

Urban upbringing and childhood trauma are both associated with psychotic disorders. However, the association between childhood urbanicity and childhood trauma in psychosis is poorly understood. The urban environment could occasion a background of social adversity against which any effect of childhood trauma increases. Also, any impact of the urban environment on likelihood of exposure to childhood trauma could be stronger in children who later develop psychotic disorder. The aim of this study was twofold: (1) to investigate whether childhood urbanicity moderates the effect of childhood trauma, in a model predicting psychotic disorder; (2) to investigate whether there is an association between the urban environment and childhood trauma and whether this is moderated by genetic liability for psychotic disorder.

**Methods:**

Patients with a diagnosis of non-affective psychotic disorder (*n* = 1119) and 589 healthy controls from the Netherlands and Belgium were studied. Childhood trauma was assessed with the Dutch version of the Childhood Trauma Questionnaire Short Form. Urban exposure was defined at four levels, considering the population density, using data from Statistics Netherlands and the equivalent database in Belgium.

**Results:**

There was a significant interaction between childhood urbanicity on the one hand and childhood trauma on the other, indicating that trauma was significantly associated with psychotic disorder, with increasing odds ratios for higher levels of childhood urbanicity. In addition, there was weak evidence that childhood urbanicity was associated with childhood trauma in the patient group: higher levels of childhood urbanicity were associated with higher trauma scores.

**Conclusion:**

The urban environment may moderate the risk-increasing effect of childhood trauma for psychotic disorder and childhood urbanicity may be a risk factor for childhood trauma in individuals who later develop psychotic disorder.

## Introduction

Urban birth and urban upbringing [1–4], and childhood trauma [5–9] are both associated with psychotic disorder. The dynamics underlying the triangular association between childhood urbanicity, childhood trauma and psychosis remains poorly understood but is of considerable interest in the prevention and management of risk given childhood exposure to adversity.

There is evidence that the incidence of childhood trauma is linked to aspects of the urban environment: neighbourhood factors, such as impoverishment and child care burden (ratio of children to adults, and the ratio of males to females), significantly increase child abuse [10]. Lower levels of social capital inherent to higher levels of urbanicity have been found to increase the odds of neglectful parenting, psychologically harsh parenting, and domestic violence [11].

**Fig. 1:**
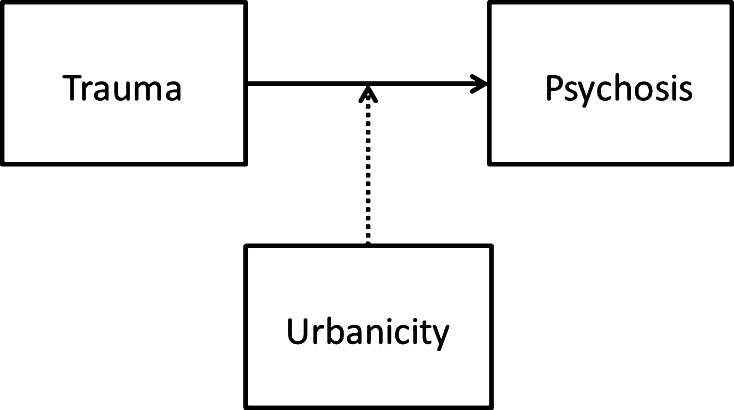
Childhood urbanicity moderates the association between childhood trauma and psychotic disorder. Childhood urbanicity is associated with social adversity, which is associated with stress. Any effect of childhood trauma on psychotic disorder in the urban environment may increase because of higher background levels of stress

**Fig. 2:**
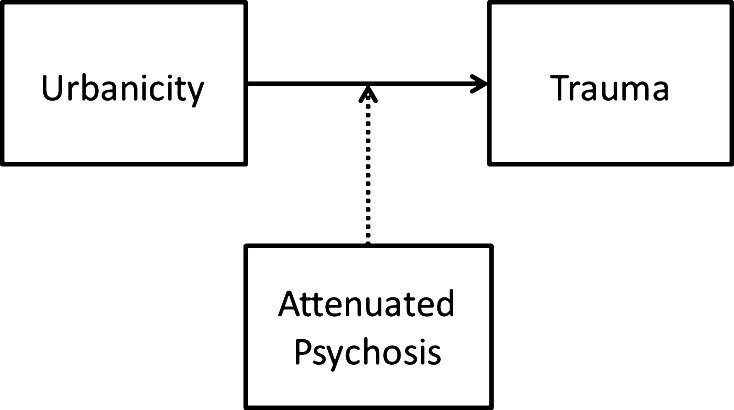
The association between childhood trauma and childhood urbanicity is moderated by genetic risk for psychotic disorder. When brought up in an urban environment with more social competition, genetic risk for psychotic disorder may increase the likelihood of exposure to childhood adversity, mediated by altered functioning of social cognition

It is not known whether urbanization moderates the effect of childhood trauma in psychosis. It may be hypothesized that the urban environment occasions a background of social adversity against which any effect of childhood trauma increases, which would indicate a model of moderation (Fig. 1). A related hypothesis is that any impact of the urban environment on the likelihood of exposure to childhood trauma is stronger in children with higher level of genetic risk for psychotic disorder (moderation by genetic risk; Fig. 2). For example, early alterations in social cognition [12–15] may increase the likelihood of exposure to childhood adversities in individuals who later develop psychotic disorder, when brought up in an urban environment. To address these issues, triangular associations between urbanicity, trauma and psychosis were examined in two directions: (1) is there evidence that childhood urban environment moderates the effect of childhood trauma on the development of psychotic disorder? and (2) is there evidence of an association between the urban environment and childhood trauma, and is this contingent on genetic liability for psychotic disorder?

## Materials and methods

### Participants

Data pertain to baseline measures of the ongoing multisite, longitudinal, naturalistic cohort study, the Dutch national Genetic Risk and Outcome in Psychosis (GROUP) project [16]. The full sample consisted of 1119 patients diagnosed with a non-affective psychotic disorder, 1057 of their siblings, 919 parents of the patients and their siblings, and 589 unrelated healthy controls subjects from the general population from the Netherlands and Belgium. Parents and siblings were not included in the current analysis.

In selected representative geographical areas of the Netherlands and (the Dutch speaking part of) Belgium, patients were identified through clinicians working in regional psychosis departments or academic centres, whose caseload was screened for inclusion criteria. Subsequently, a group of patients with a clinical diagnosis of non-affective psychotic disorder presenting consecutively at these services either as outpatients or inpatients were recruited for the study. Over 30 interviewers per site were trained for administering the assessments. The interviewers consisted of research assistants, psychologists, psychiatrists, nurses and PhD students. Before the start of the study, all interviewers met for 3 days of training workshops at one site (Utrecht), to practise the assessments of all measures used in the GROUP project.

Assessments took place at one of the participating regional psychosis departments or academic centres in and around Amsterdam, Utrecht, Groningen and Maastricht. If participants were unable to visit the institute, assessments at home were offered.

Inclusion criteria were the following: (1) age range 16–50 years; (2) diagnosis of non-affective psychotic disorder; (3) sufficient command of the Dutch language; and (4) a first contact with mental health facilities within the last 10 years. Controls had no first-degree relative with a psychotic disorder as established by the Family Interview for Genetic Studies [17] with the control as informant. Diagnosis was based on the DSM-IV-TR criteria [18] assessed with the Comprehensive Assessment of Symptoms and History interview [19] or Schedules for Clinical Assessment for Neuropsychiatry version 2.1 [20].

The study was approved centrally by the Ethical Review Board of the University Medical Centre Utrecht. Written informed consent was obtained from all subjects after they (1) read a document with detailed information about the nature and possible consequences of the study; (2) had verbally discussed any possible concerns with the researcher; and (3) had provided clear indication that they had understood the procedure. In the Netherlands, adult patients with mental illness are considered participating citizens who have the right to make independent informed decisions including the autonomous decision to participate in research; therefore, consent of relatives was not sought.

### Childhood trauma

Childhood trauma was assessed with the Dutch version of the Childhood Trauma Questionnaire Short Form (CTQ) [21]. The short CTQ consists of 25 items rated on a 5-point Likert scale (1 = never true to 5 = very often true) enquiring about traumatic experiences in childhood. Five types of childhood maltreatment were assessed: emotional, physical and sexual abuse, and emotional and physical neglect, with five questions covering each type of trauma. The mean score for all 25 items was used as the total trauma rating (CTQ total). CTQ data were missing for 454 persons (27 % missing data, see below). The trauma variable was dichotomized a priori into high trauma and low trauma. As in previous analyses, the cutoff was defined as the 80th percentile of scores for the healthy comparison subjects [8]. Subjects with a score of 1.52 or higher were in the high-trauma group.

### Level of urbanicity

A historical population density record was generated for each municipality from 1930 onwards using historical data from Statistics Netherlands and the equivalent database in Belgium [22, 23]. When data were not available, missing data were calculated by linear extrapolation between two subsequent time points. When historical names of municipalities disappeared from historical records (e.g. due to city mergers), the available data from the agglomerate city were used. Subjects were asked to describe where they had lived at birth, between ages 0 and 4 years; 5 and 9 years; 10 and 14 years; 15 and 19 years; 20 and 39 years; 40 and 59 years; and 60+ up to the actual age. This resulted in a number of records for each subject, containing locations by age period. For each of these records, we computed the average population density (by square kilometre, excluding water) of the municipality for the matching periods. Average population density over the period was categorized in accordance with the Dutch CBS urbanicity rating (1 = <500/km^2^; 2 = 500–1000/km^2^; 3 = 1000–1500/km^2^; 4 = 1500–2500/km^2^; 5 = 2500+/km^2^). The periods 0–4 years, 5–9 years and 10–14 years were collapsed to produce average urbanicity exposure between 0 and 14 years, rounded to the nearest whole number. Categories 3 and 4 were combined into a single category, because numbers of participants in these two categories were small compared to the other categories, this resulted in 4 categories (1 = <500/km^2^; 2 = 500–1000/km^2^; 3 = 1000–2500/km^2^; 4 = 2500+/km^2^). The latter was used as the primary variable reflecting childhood urbanicity exposure in the analyses. Urbanicity data were missing for 148 persons (9 % missing data).

### Intelligence quotient

To estimate IQ, we used four subtests of the Dutch version of the Wechsler Adult Intelligence Scale-III (WAIS-III) [24] consisting of the subtests ‘Information’, ‘Block Design’, ‘Digit Symbol Coding’ and ‘Arithmetic’. The combination of these four subtests has been shown reliable for estimating IQ in schizophrenia patients and controls [25].

### Statistical analyses

All analyses were performed using Stata 12 [26]. To test the first hypothesis (childhood urbanicity moderates the association between childhood trauma and psychotic disorder), logistic regression models were fitted with group (group status defined as: control or patient) as the dependent variable and childhood trauma and childhood urbanicity as independent variables and age and sex as possible confounders. The two-way interaction between urbanicity (entered as a linear variable and dummy variable in separate models) and trauma (entered as a linear variable and a dichotomous variable in separate models) was added to the model to test for moderation. Interactions were evaluated by Wald test [27]. In the case of significant interaction, odds ratios of trauma per category of urbanicity were calculated using the model containing the interactions, applying the Stata LINCOM routine.

To test the second hypothesis (the level of urbanization may be more strongly associated with childhood trauma in children who later develop psychotic disorder), linear regression models were fitted with the trauma rating as the dependent variable and group and childhood urbanicity as independent variables, adding age and sex as possible confounders. Because of the uneven distribution of CTQ total, a qq-plot of the regression residuals was made, to check for possible violation of model assumptions. To model the possible modifying effect of childhood urbanicity on measures of proxy genetic risk, the two-way interaction between urbanicity (entered as a linear variable and dummy variable) and group was added to the model. Again, interactions were evaluated by Wald test [27]. In the case of significant interaction, effect sizes were calculated by combination of effects from the model containing the interactions using the Stata LINCOM routine. Childhood trauma and childhood urbanicity scores were not available for all participants; therefore, 696 patients and 467 controls were included in the final analyses.

To account for missing values in CTQ total, sensitivity analyses were performed using the Stata multiple imputation suite of commands (mi). Missing values were assumed to be missing at random (MAR) and sex, group and educational level were used to impute missing values for trauma. All interaction models were imputed ten times.

## Results

### Participants, descriptives and main effects

The total sample consisted of 1119 patients with a diagnosis of non-affective psychotic disorder and 589 control subjects. Sociodemographic and clinical characteristics of the final sample are summarized in Table 1. Sixteen patients had a diagnosis of depression of anxiety disorder; these patients were retained in the analyses given that they had a past clinical diagnosis of psychotic disorder on the basis of which they had been selected into the sample. The patient group had a higher proportion of men and displayed lower IQ than the control group. Multilevel linear regression analysis showed that CTQ total was significantly higher in the patient group compared to the control group. Childhood urbanicity did not differ between the two groups (patients: *B* = 0.02, *p* = 0.83). Childhood urbanicity was not significantly associated with childhood trauma in the total sample (*B* = 0.02, *p* = 0.06).

**Table 1:** Subject demographics

	Patients (*n* = 1119)	Controls (*n* = 589)
Age	27.6 ± 8.0	30.4 ± 10.6
Sex *n* (%), male	852 (76.1)	269 (45.6)
Ethnicity		
Caucasian *n* (%)	857 (76.6)	530 (90.0)
Other n (%)	262 (23.4)	59 (10.0)
Childhood urbanicity^a^	2.7 ± 1.6	2.6 ± 1.6
CTQ	1.6 ± 0.5	1.3 ± 0.4
Diagnosis *n* (%)		
No diagnosis on axis I	–	536 (91.0)
Schizophrenia-related disorder	792 (71.4)	–
Schizoaffective disorder	120 (10.8)	–
Brief psychotic disorder	33 (3.0)	–
Delusional disorder	22 (2.0)	–
Substance-induced psychotic disorder	5 (0.5)	–
Psychotic disorder NOS	118 (10.6)	–
Psychotic disorder due to medical condition	1 (0.1)	–
Mood disorder	16 (1.4)	52 (8.8)
Delirium	1 (0.1)	–
Anxiety-related disorder	–	1 (0.2)
Substance-related disorder	1 (0.1)	–

*CTQ* Childhood Trauma Questionnaire, range 1–5

^a^Five levels of urbanicity/population density 1 = <500 inhabitants/km^2^; 2 = 500–1000 inhabitants/km2; 3 = inhabitants 1000–1500/km^2^; 4 = inhabitants 1500–2500/km^2^; 5 = 2500+/km

### Childhood urbanicity moderates the association between childhood trauma and psychosis

The two-way interaction term childhood trauma × childhood urbanicity (both variables entered as linear variables) was significant in the case–control model (*χ* ^2^ = 7.06, *df* = 1, *p* = 0.01). High CTQ scores were associated with psychotic disorder with increasing odds ratios for higher levels of childhood urbanicity (Table 2). The odds ratios increased roughly linear with higher levels of childhood urbanicity.

**Table 2:** Association between high trauma scores and patient status across levels of childhood urbanicity

	Odds ratio	95 % CI	*p*
Urbanicity 1	2.76	1.71–4.46	<0.001
Urbanicity 2	4.12	2.27–7.45	<0.001
Urbanicity 3	5.61	2.78–11.33	<0.001
Urbanicity 4	5.66	2.87–11.16	<0.001

The trauma variables were dichotomized a priori into high trauma and low trauma. The cutoff was defined as the 80th percentile of scores for the healthy comparison subjects

Four levels of urbanicity/population density 1 = <500 inhabitants/km^2^; 2 = 500–1000 inhabitants/km^2^; 3 = inhabitants 1000–2500/km^2^; 4 = 2500+/km

*CI* confidence interval

### Association between urbanicity and trauma and moderation by genetic risk

The two-way interaction term group × childhood urbanicity was statistically significant in the models analysing CTQ total (*χ* ^2^ = 5.79, *df* = 1, *p* = 0.02). The direction of effects in the patients and in the controls appeared to be opposite, although not significantly so and may reflect a chance finding. In the patients, CTQ total score increased with higher levels of childhood urbanicity (*B* linear trend = 0.02, *p* = 0.10). In the control group, increasing levels of childhood urbanicity were associated with lower CTQ total scores (*B* Linear trend = −0.03, *p* = 0.08) (Table 3).

**Table 3:** Mean CTQ total scores by group and level of childhood urbanicity

Urbanicity	Patients			Controls		
	*N*	Mean CTQ (SD)	*B* (*p*)	*N*	Mean CTQ (SD)	*B* (*p*)
1	239	1.55 (0.46)		174	1.40 (0.39)	
2	157	1.69 (0.54)	0.13 (<0.01)	107	1.36 (0.40)	−0.03 (0.60)
3	144	1.58 (0.49)	0.02 (0.64)	91	1.26 (0.26)	−0.12 (0.04)
4	156	1.65 (0.52)	0.10 (0.03)	95	1.32 (0.29)	−0.07 (0.21)
*B* linear trend			0.02 (0.10)			−0.03 (0.08)

Four levels of urbanicity/population density 1 = <500 inhabitants/km^2^; 2 = 500–1000 inhabitants/km^2^; 3 = inhabitants 1000–2500/km^2^; 4 = 2500+/km

*CTQ* childhood trauma questionnaire, *SD* standard deviation, *B* the regression coefficients from multilevel linear regression analyses, adjusted for age and sex

### Sensitivity analysis

After imputation the interaction between childhood trauma and childhood urbanicity in the model, predicting case–control status was no longer statistically significant by conventional alpha, but was still suggestive for interaction (*χ* ^2^ = 3.58, *df* = 1, *p* = 0.06). The interaction between childhood urbanicity and group in the model predicting CTQ total also was reduced but still suggestive for interaction (*χ* ^2^ = 3.47, *df* = 1, *p* = 0.06).

## Discussion

The association between childhood trauma, childhood urbanicity and psychotic disorder was examined. Childhood urbanicity appeared to moderate the association between childhood trauma and psychosis: the risk-increasing effect of childhood trauma for psychotic disorder was stronger for higher levels of childhood urbanicity. Further, childhood urbanicity was at trend level associated with childhood trauma: higher levels of childhood urbanicity were associated with higher trauma scores, but only in the patient group.

The finding that childhood urbanicity moderates the effects of childhood trauma is new, but also in line with previous findings concerning other risk factors. Kuepper and colleagues [28] suggested that urbanicity moderates the effect of cannabis use. The effect of cannabis use on psychotic symptoms was stronger in individuals who grew up in an urban environment, compared to those who grew up in a rural environment. In addition, the co-occurrence of multiple environmental risk factors for psychosis in persons with low-grade psychotic experiences, including childhood trauma and childhood urbanicity, has been associated with persistence of psychotic symptoms [29].

Childhood urbanicity may moderate the effect of childhood adversity by creating additional exposure to stress [30]. Childhood trauma has been associated with alterations of the mechanisms subserving stress regulation and with altered functioning of the hypothalamic–pituitary–adrenal axis (HPA axis) [31, 32]. Higher levels of social isolation [33] and social defeat [34] in urban areas can lead to higher background levels of stress, which may have an additional effect on the HPA axis. Altered functioning of the hypothalamic–pituitary–adrenal axis may impact dopaminergic signalling, and may lead to sensitization of mesolimbic dopamine neurons in early adulthood, contributing to onset of psychosis [32].

Childhood abuse and childhood neglect are two different aspects of childhood trauma. In post hoc analyses, childhood abuse and child neglect were examined separately in the model predicting psychotic disorder. Childhood urbanicity appeared to strengthen the association between childhood neglect and psychotic disorder more than the association between childhood abuse and psychotic disorder. Childhood neglect has been linked to the absence of social support, social isolation, and being in a financially disadvantaged position, all predicting suboptimal parenting [28–30]. Higher levels of perceived social isolation in urban areas [26] and greater exposure to social “defeat” occasioned by higher levels of competition in cities [27] are proposed mechanisms for the association between childhood urbanicity and psychotic disorders. It is conceivable that such disadvantages occasioned by the urban environment intensify any effects of childhood neglect.

Previous studies reported an association between childhood trauma and childhood urbanicity in the non-psychotic population. Drake et al. [35] found that especially childhood neglect, and not psychological or sexual abuse, was most powerfully associated with urban neighbourhood poverty. Breakdown of community social control and organization is associated with an increase of child maltreatment [36]. Neighbourhood characteristics seem to have an effect on parents’ level of stress and personal control, which in turn is a risk factor for physical child abuse and neglect [37, 38]. Also, the protective influence of social support on parenting behaviour diminishes in poor and dangerous neighbourhoods [39]. In our total study sample, the association between childhood trauma and childhood urbanicity was in the same direction, although it was only significant at trend level. An explanation of the absence of a significant association could be that the number of participants in the higher urbanicity levels was relatively small compared to the lower urbanicity levels, resulting in low power. These results have to be interpreted carefully since more than 50 % of the study subjects were patients, which may have biased these results. In a different population-based sample, this association may be even less or absent.

Our results suggest that individuals who later develop psychotic disorder could be more susceptible to exposure to childhood trauma when growing up in an urban environment. In controls, no such trend was apparent. Because childhood trauma [9] and childhood urbanicity [40] both appear to be substantial risk factors for psychotic disorders, growing up in a city and the subsequently higher risk for childhood trauma (gene environment co-association) will even further increase the risk of psychotic disorder.

One possible explanation for these findings is that impaired social cognition combined with living in an urban environment, with higher levels of social competition, could increase the likelihood to experience interpersonal adversity and, possibly, childhood trauma. From meta-analytic evidence, it is known that social cognition is impaired in psychotic disorder [12]. In addition, social cognition is not only altered in psychotic disorder, but also in persons at genetic and clinical high risk for psychosis [13–15]. Therefore, alterations in social cognition may represent a candidate explanation for the association between urbanicity and trauma in persons at higher genetic risk for psychotic disorder. This explanation needs to be tested in future research, just as replication of the current findings is necessary. It is also conceivable that the association between childhood trauma and social cognition is inverted: experience of childhood trauma may result in impaired social cognition. If this is the case then impaired social cognition could be on the causal pathway from childhood trauma to psychotic disorder. This concept is supported by a study showing deficits in theory of mind after deprivation during childhood [41].

The results may be compatible with childhood trauma partially mediating the association between childhood urbanicity and psychotic disorder. As this data set did not reveal an association between childhood urbanicity and psychotic disorder, the possibility of mediation by childhood trauma could not be tested. There was an association between childhood trauma and psychotic disorder in the study sample and therefore moderation by childhood urbanicity could be explored. The hypothesis of childhood urbanicity (partially) mediating the association between childhood trauma and psychotic disorder also could not be tested because of the absence of an association between urbanicity and psychotic disorder. Moreover, childhood trauma contributing to childhood urbanicity has less face validity than childhood urbanicity contributing to risk of childhood trauma.

## Limitations

First, our findings must be interpreted with caution because the effect sizes were relatively small. Second, 27 % of the trauma data was missing. However, sensitivity analyses imputing missing data showed similar results. Third, the cross-sectional and retrospective design of the present data analysis does not allow us to establish a causal link between childhood urbanicity, childhood trauma and psychosis. From these data, we can only conclude that there is an association, indicating that causality has to be further investigated in future research. Further, case–control studies are sensitive to selection bias. Participation of the control subjects could have been influenced by self-selection of persons with higher levels of childhood trauma. However, if this were the case, the analyses may be considered conservative. If the opposite were true and control subjects with a history of childhood trauma were less likely to participate, there may be an overestimation of the effect. Thus, data have to be interpreted with awareness of a possible selection bias. Fourth, the childhood trauma questionnaire is a retrospective and self-reported questionnaire. Nevertheless, the childhood trauma questionnaire is reliable in assessing trauma accurately [42, 43]. Finally, the participants grew up in the Netherlands and Belgium which can be described as relatively safe and well-developed countries; in other counties, urban–rural discrepancies may be more prominent. However, if this were true, effect sizes in other countries would be more substantial.

## Conclusion

The results substantially support that childhood urbanicity moderates the association between childhood trauma and psychotic disorder, and tentatively indicate that childhood urbanicity may be a risk factor for childhood trauma in individuals with a high genetic liability for psychotic disorder. Future research is needed to replicate these findings and also more research on the risk-increasing mechanisms, e.g. evaluating social cognition, in urban environments is required.

## Appendix

Genetic Risk and Outcome of Psychosis (GROUP) Investigators: Richard Bruggeman^a^, WiepkeCahn^b^, Lieuwe de Haan^c^, René Kahn^b^, Carin Meijer ^c^, Inez Myin-Germeys^d^, Jim van Os^d,e^, DurkWiersma^a^

^a^University Medical Center Groningen, Department of Psychiatry, University of Groningen, The Netherlands, ^b^University Medical Center Utrecht, Department of Psychiatry, Rudolf Magnus Institute of Neuroscience, The Netherlands, ^c^Academic Medical Centre University of Amsterdam, Department of Psychiatry, Amsterdam, The Netherlands, ^d^Maastricht University Medical Centre, South Limburg Mental Health Research and Teaching Network, EURON, Maastricht, The Netherlands, ^e^King’s College London, King’s Health Partners, Department of Psychosis Studies, Institute of Psychiatry, London, United Kingdom.
